# KR158 Spheres Harboring Slow-Cycling Cells Recapitulate High-Grade Glioma Features in an Immunocompetent System

**DOI:** 10.3390/cells13110938

**Published:** 2024-05-29

**Authors:** Avirup Chakraborty, Changlin Yang, Jesse L. Kresak, Aryeh J. Silver, Diana Feier, Guimei Tian, Michael Andrews, Olusegun O. Sobanjo, Ethan D. Hodge, Mia K. Engelbart, Jianping Huang, Jeffrey K. Harrison, Matthew R. Sarkisian, Duane A. Mitchell, Loic P. Deleyrolle

**Affiliations:** 1 Adam Michael Rosen Neuro-Oncology Laboratories, Department of Neurosurgery, University of Florida, Gainesville, FL 32608, USAaryehjsilver@gmail.com (A.J.S.);; 2 Preston A. Wells Jr. Center for Brain Tumor Therapy, University of Florida, Gainesville, FL 32608, USA; 3 Department of Pathology, Immunology and Laboratory Medicine, University of Florida, Gainesville, FL 32610, USA; 4 Department of Surgery, University of Florida, Gainesville, FL 32610, USA; 5 College of Dental Medicine, Nova Southeastern University, Fort Lauderdale, FL 33314, USA; 6 Department of Pharmacology and Therapeutics, University of Florida, Gainesville, FL 32603, USA; 7 Department of Neuroscience, McKnight Brain Institute, University of Florida, Gainesville, FL 32610, USA

**Keywords:** glioblastoma, cancer stem cells, slow-cycling cells, tumor heterogeneity, immunocompetent murine model

## Abstract

Glioblastoma (GBM) poses a significant challenge in clinical oncology due to its aggressive nature, heterogeneity, and resistance to therapies. Cancer stem cells (CSCs) play a critical role in GBM, particularly in treatment resistance and tumor relapse, emphasizing the need to comprehend the mechanisms regulating these cells. Also, their multifaceted contributions to the tumor microenvironment (TME) underline their significance, driven by their unique properties. This study aimed to characterize glioblastoma stem cells (GSCs), specifically slow-cycling cells (SCCs), in an immunocompetent murine GBM model to explore their similarities with their human counterparts. Using the KR158 mouse model, we confirmed that SCCs isolated from this model exhibited key traits and functional properties akin to human SCCs. KR158 murine SCCs, expanded in the gliomasphere assay, demonstrated sphere forming ability, self-renewing capacity, positive tumorigenicity, enhanced stemness and resistance to chemotherapy. Together, our findings validate the KR158 murine model as a framework to investigate GSCs and SCCs in GBM pathology, and explore specifically the SCC–immune system communications, understand their role in disease progression, and evaluate the effect of therapeutic strategies targeting these specific connections.

## 1. Introduction

GBM, the most common malignancy of the central nervous system, continues to present a significant challenge in oncology due to its aggressive nature and resistance to conventional therapies. The involvement of CSCs in GBM, especially in driving resistance to treatment and tumor relapse, makes it a priority to fully understand the mechanisms regulating this phenotype or cellular state. Extensive research has highlighted the multifaceted role of these cells, demonstrating their contribution to tumor initiation, therapeutic resistance, and recurrence. Their distinctive properties, notably their long-term self-renewal capacity and ability to generate a large number of progenies, underscore their influential role in driving the aggressive behavior of GBM.

Tumor heterogeneity is also a recognized major determinant of treatment failure and recurrence. Understanding the key mechanisms driving this heterogeneity is paramount to improving our understanding of the complex biology of GBM and designing effective new therapies. CSCs are key to this heterogeneity and comprise diverse heterogeneous populations with multiple factors, both intrinsic and extrinsic, contributing to their phenotypic and functional diversity [1,2,3,4,5]. Findings from our laboratory and others described CSCs in several malignancies [6,7,8,9,10,11,12,13,14,15,16,17], including GBM [15,16,17,18,19,20,21,22,23,24], in which the existence of a subpopulation of CSCs, namely slow-cycling cells (SCCs), has gained attention due to their resistance to standard-of-care therapies and their role in GBM progression and recurrence. Despite their clinical relevance, effective strategies to eliminate these cells remain elusive. Hence, it is imperative to advance our understanding of their biology and identify vulnerabilities that can be exploited for therapeutic advancements.

The evolving comprehension of the role of the immune system within the GBM landscape has reshaped perspectives on the study of the TME, with recognition of immune responses, or lack thereof, emerging as a critical factor influencing clinical outcomes. This expanding understanding has prompted investigations into the dynamic interactions between cancer cells, especially CSCs and SCCs, and immune cells within the GBM microenvironment. Therefore, the complexities of the TME, comprising diverse cell types and their intricate interactions, represent a focal point in unraveling GBM trajectory and treatments. 

Developing and using preclinical GBM models adequately representing the TME, specifically mirroring its complexity, morpho-pathological characteristics, and immune landscape, is a prerequisite to integrate the dynamics of the different cellular components and their collaborative or antagonistic interactions. Deciphering the intricate relationships between tumor cells, e.g., SCCs, and the immune system and investigating the impact of tumor heterogeneity on the immune milieu has the potential to unveil pivotal mechanisms dictating disease progression and treatment responses. 

One of the well-studied and -used immunocompetent murine models of high-grade glioma to address these issues is KR158 [25]. This model of glioma is driven by the mutation of two tumor-suppressor genes, NF1 and TP53 [25]. Despite its relevance, this model has limitations in recapitulating human GBM pathology, specifically due to its tendency to mirror grade 3-like gliomas rather than grade 4-like tumors. Although orthotopic transplantation results in approximately 60% of malignant lesions displaying high-grade glioma features, such as multifocal lesions, varying cellularity, high mitotic index, and microscopic necrotic foci, it lacks extensive infiltration and the distinctive pseudopalisading around necrotic areas, thus falling short of classification as grade 4, or glioblastoma.

The gliomasphere assay was designed to enrich glioma stem cells capable of forming tumors that reproduce all key morphological traits of grade 4 glioma [26,27]. In our study, we leveraged this assay to culture murine KR158 cells, aiming to isolate cellular populations proficient in recapitulating these distinctive characteristics. Our findings demonstrate that KR158 cells expanded under these conditions produce tumors resembling GBM when transplanted intracranially. These results support the efficacy of this assay in more closely recapitulating the human disease as compared to KR158 cells cultured in adherent monolayer serum-containing conditions.

Our previous research established the existence of a metabolic diversity within the microenvironment of human GBM, which includes fast- and slow-cycling cells with distinct metabolic features [1,5,16,19]. Moreover, SCCs isolated from human GBM tumors have demonstrated increased stemness, enhanced invasion, and drug resistance, pointing to their role in tumor progression and recurrence [16,19,20]. The objective of this study was also to validate the slow-/fast-cycling cell paradigm in an immunocompetent model of glioma with the long-term goal of exploring the connections between the intratumor heterogeneity and the immune landscape in these tumors. This work showed that in the KR158 mouse model of glioma [25], SCCs share phenotypic and functional traits of their human counterparts [16,19,20]. We found that KR158 SCCs are tumorigenic, generate tumors displaying key features of GBM, and exhibit a greater stemness phenotype and specific metabolic signature and enhanced resistance to chemotherapy. Together, our results validate the potential use of this model for a comprehensive exploration of the intricate connections between CSCs, especially SCCs, the immune system, and the TME in GBM. This exploration will emphasize their profound implications for disease understanding and the development of innovative therapeutic strategies.

## 2. Materials and Methods 

### 2.1. Murine Glioma Cell Line

The murine KR158 (wildtype or expressing luciferase [KLuc]) glioma cell line was kindly provided by Dr. Tyler Jacks, Massachusetts Institute of Technology, Cambridge, MA, USA [25]. These cells were cultured both in serum-free gliomasphere assay conditions as floating spheres and in standard adherent monolayer and serum-containing conditions as described below. CT-2A cells [28,29,30] were also cultured in the gliomasphere assay for SCC identification. 

### 2.2. Gliomasphere Assay

Under serum-free conditions, the cells were cultured at 37 °C in the presence of 5% CO_2_ in NeuroCult NS-A proliferation solution with 10% proliferation supplement (STEMCELL Technologies, Vancouver, BC, Canada; Cat#05750 and #05753) supplemented with 20 ng/mL mouse EGF (R&D Systems, Minneapolis, MN, USA, Cat#2028-EG) and 10 ng/mL human FGF2 (R&D Systems, Minneapolis, MN, USA; Cat#233-FB/CF), which stimulates the proliferation of murine cells [31]. Human and murine FGF receptors are highly conserved and show a 95% homology [32]. We also included 10 ng/mL of heparin (Sigma-Aldrich, St. Louis, MO, USA, Cat#H3149), as it regulates FGF2 activity and enhances specificity and affinity toward the FGF2 receptor, thereby modulating the transduction cascade and stimulating cell proliferation [33,34,35]. Cultures were maintained in 1% antibiotic-antimycotic (Life Technologies, Carlsbad, CA, USA, Cat#15240062). Upon reaching a diameter of about 150 µm, gliomaspheres were enzymatically digested using Accutase (StemCell Technologies, Vancouver, BC, Canada, Cat#07920) for 15 min at 37 °C. Subsequently, the cells were washed, counted, and replated in a fresh serum-free complete medium for further expansion and downstream experimentation.

### 2.3. Classical Adherent and Serum-Containing Cell Cultures

The cells were cultured at 37 °C in the presence of 5% CO_2_ in DMEM (Gibco, NY, USA Cat#11965092) and supplemented with 10% FBS (Avantor, PA, USA, Cat#89510186) and 1% antibiotic-antimycotic (Life Technologies, Waltham, MA, USA, Cat#15240062). Upon reaching 90% confluency, the cells were enzymatically digested using Accutase (StemCell Technologies, Vancouver, BC, Canada, Cat#07920) for 15 min at 37 °C and were subsequently washed and replated in fresh complete medium.

### 2.4. Scratch-Wound Assay and Time-Lapse Imaging

Cells that were expanded in the gliomasphere assay for several passages were plated in a 35 mm time-lapse imaging dish. The attachment of the cells for this assay was stimulated by the addition of 10% FBS. Imaging was performed on an inverted Zeiss Axio-Observer D1 microscope (Oberkochen, Baden-Württemberg, Germany). Imaging dishes were secured onto a stage-top incubation system and maintained in a humid chamber at 37 °C and 5% CO_2_ using a Tokai Hit System (Bala Cynwyd, PA, USA). Image acquisition and processing were performed using the Zeiss software (ZEN 2012 Blue edition v1.1.2.0). Images were acquired every 10 min and movies were exported at 5 frames per second.

### 2.5. Intracranial Implant, Tumor Growth Monitoring and Survival Analysis 

To compare the tumor-generating capabilities of the (i) total unsorted KLuc cells grown as gliomaspheres against those grown as adherent cells and (ii) total unsorted KLuc, fast-cycling cells (FCCs) and SCCs grown in the gliomasphere assay, the tumor cells were intracranially implanted in immunocompetent 7- to 15-week-old C57BL/6 mice following NIH and institutional (IACUC) guidelines and regulations for animal care and handling. The mice colonies were maintained at the University of Florida’s animal facility. These mice were intracranially implanted with 2 µL of cell suspension containing 10,000 live KLuc cells using a sterile 5 mL Hamilton syringe fitted with a 25-gauge needle into the striatum using a stereotactic apparatus. The injection coordinates were 2.0 mm lateral to the bregma at a depth of 3.0 mm below the dura mater, as previously described [16,19,36]. Longitudinal monitoring of the tumor volume was performed using an IVIS Spectrum imaging system (Xenogen, Alameda, CA, USA) measuring bioluminescence related to luciferase activity. The animals were also monitored for any neurological signs affecting their quality of life. When symptoms including ataxia, lethargy, seizures, or paralysis were observed, the mice were sacrificed. 

### 2.6. Isolation of Fast- and Slow-Cycling Cells

SCC and FCC populations grown as glioma spheres were identified and isolated based on their proliferation rate, which was accessed based on their ability to retain carboxyfluorescein succinimidyl ester (CFSE) [Cat#c1157] or CellTrace™ Violet (CTV) [Cat#c34571], (Invitrogen, Waltham, MA, USA), as described previously [16,17,19] by studying the CFSE/CTV fluorescence intensity decay rate over time, as measured using flow cytometry. Four to seven days post labeling, these cells were grouped as CFSE/CTV^High^, top 10% (referred to as SCCs), and CFSE/CTV^Low^, bottom 10% (referred to as FCCs). This gating strategy allowed us to isolate the functional and phenotypic extremes with similar-sized populations, homogenizing for sorting time and addressing potential issues related to fluorescence-activated cell sorting (FACS)-induced metabolic stress. The utilization of these extreme fractions from the proliferation spectrum ensured a clear and distinct separation of FCCs and SCCs based on cell cycle kinetics. All experiments were promptly conducted following the FACS of SCC and FCC populations.

### 2.7. Short Tandem Repeat (STR) Analysis

Idexx STR CellCheck^TM^ 19 was implemented to compare the genetic profile of KR158 cells expanded in the gliomasphere assay or cultured with serum as a monolayer. 

### 2.8. Quantification of Secreted Lipocalin-2 Using ELISA 

To assess lipocalin-2 (LCN2) secretion from SCCs and FCCs, we utilized an enzyme-linked immunosorbent assay (ELISA). Following cell isolation procedures outlined previously, we seeded 100,000 SCCs and FCCs (*n* = 6) and cultured them under serum-free conditions for 48 h, as described above. Conditioned media were then collected, and LCN2 levels were measured using a commercially available ELISA kit (R&D Systems, Minneapolis, MN, USA, Cat#DY1857) as per the manufacturer’s instructions. Briefly, the collected media was incubated with LCN2-specific capture antibodies, followed by detection antibody conjugated to Streptavidin-HRP B. The subsequent addition of the substrate solution allowed for quantification by measuring optical density at 450 nm, with readings at 540 nm subtracted for correction. This approach facilitated the comparison of LCN2 secretion dynamics between SCCs and FCCs, offering insights into their respective secretory profiles.

### 2.9. Immunocytochemistry

KR158 cells were cultured in gliomasphere assay conditions as previously described and stained with 5 μM of CellTrace™ Violet (CTV, Thermo Fisher Scientific, Waltham, MA, USA, Cat#C34557) to identify the subpopulations of SCCs and FCCs. Approximately five days post-CTV loading, the cells were seeded onto poly-D-lysine-coated coverslips. Following media aspiration, the cells were fixed with 4% paraformaldehyde (Thermo Scientific; Waltham, MA, USA). After washing, cells were permeabilized using 0.1% TritonX100 (Sigma-Aldrich, St. Louis, MO, USA), followed by antigen blocking using 1% BSA (Sigma-Aldrich, St. Louis, MO, USA). Subsequently, fixed and permeabilized cells were stained with an antibody against Lipocalin 2 (1/50; R&D Systems, Minneapolis, MN, USA; Cat#AF1757), followed by incubation with a donkey anti-goat AF488 conjugated secondary antibody (1/500; Thermo Fisher Scientific, Waltham, MA, USA, Cat#A-11055). Finally, cells were mounted using ImmunoHistoMount™ Mounting Medium (Abcam, Cambridge, UK, Cat#ab104131), and stained coverslips were examined using an inverted Zeiss AxioObserver D1 microscope (Zeiss, Oberkochen, Baden-Württemberg, Germany). This allowed us to study the protein expression of Lipocalin 2 (LCN2) in the CTV retaining and diluting subpopulation of cells within the KR158 cell line cultured under gliomasphere assay conditions.

### 2.10. Flow Cytometric Analysis

KR158 cells cultured under gliomasphere assay conditions were stained with 5μM of CellTrace™ Violet (CTV, Thermo Fisher Scientific, Waltham, MA, USA, Cat#C34557) for determining the subpopulation of SCCs and FCCs. On day 4–5 post-CTV loading, the cells were labeled with live/dead dye (1:1000; Cat#L34976A Invitrogen, Waltham, MA, USA), followed by immunostaining to assess the relative target protein expression within the SCC and FCC subpopulations. Specifically, cells were subjected to staining with antibodies against the stemness markers Sox2 (0.25 μg/10^6^ cells/100 μL; R&D Systems, Minneapolis, MN, USA; Cat#MAB2018) with a goat anti-mouse AF647 conjugated secondary antibody (10 μg/mL; Cat#11008 Invitrogen, Waltham, MA, USA) and CD44 (0.25 μg/10^6^ cells/100 μL; Biolegend, San Diego, CA, USA; Cat#103008); the migration marker CXCR3 (0.25 μg/10^6^ cells/100 μL; Biolegend, San Diego, CA, USA; Cat#126515); and the lipid trafficking protein Fabp3 (1:50; Cat#PA5-13461 Invitrogen, Waltham, MA, USA) with a goat anti-rabbit AF647 conjugated secondary antibody (10 μg/mL; Cat#A10523 Invitrogen, Waltham, MA, USA). Flow cytometric acquisition was then conducted at the Flow Cytometry and Imaging Core, UF Interdisciplinary Center for Biotechnology Research (ICBR; RRID:SCR_019119) using a BD FACSymphony A3 (BD Biosciences, Franklin Lakes, NJ, USA) instrument and analysis performed using FlowJo 10.10.0 to quantify the levels of different proteins.

### 2.11. Brain Tumor Tissue Processing

Upon reaching the defined humane endpoint or upon reaching the designated study time point, the mice were euthanized, and the tumor tissues were resected and frozen in OCT or preserved in paraffin. The preserved tissues were then sectioned into 5–8 µm slices and placed on slides. In situ tumor formation with classical morphological features of GBM including infiltration, nuclear pleomorphism with mitotic figures, and pseudopalisading necrosis was confirmed using hematoxylin and eosin staining of the paraffin-embedded sections. Further, we determined the invasiveness of the luciferase-expressing tumor cells by staining the frozen OCT-embedded sections with anti-firefly luciferase antibody (1:500 Abcam, Cambridge, UK Cat#21176 respectively) and a goat anti-rabbit AF488 conjugated secondary antibody (1:1000; Cat#11008 Invitrogen, Waltham, MA, USA).

### 2.12. Live/Dead Assay

Propidium iodide (PI) incorporation assay using flow cytometry: To determine the impact of temozolomide (TMZ) on the viability of the SCCs, FCCs, and the total unsorted tumor cells, we performed an in vitro PI incorporation assay. The cells were stained with CTV and expanded for 5–7 days before being exposed to a dosage of 400 µM of TMZ for 48 h. Their viability was assessed using PI staining (Thermo Scientific; Waltham, MA, USA). Cell staining was performed with the method as recommended by the manufacturer’s manual. The incorporated PI was quantified using flow cytometry (BD LSRII; BD Biosciences Franklin Lakes, NJ, USA). The cells were gated with respect to the intensity of CTV and identified as SCCs and FCCs as described above. PI positive cells were indicative of dead cells. The value of PI positive cells represents the mean of three independent experiments. 

### 2.13. CyQUANT Direct Cell Proliferation Assay

The cytotoxic effects of TMZ at different dosages (1, 2, and 3 mM) was further assessed by using the fluorescence-based CyQUANT cell proliferation assay (Thermo Fisher Scientific Waltham, MA, USA, Cat#C7026). The assay measures proliferation and membrane integrity, which is another measure of cell health. Sorted SCCs, FCCs, and unsorted tumor cells were plated at 80,000 cells per well in 96-well plates and exposed to TMZ. After 48 h, CyQUANT binding dye was added to each well and incubated for 30 min at 37 °C before being quantified using BiotekTM Cytation^TM^ 3 Cell Imaging Multi-Mode Reader. The value of relative cell proliferation represents the mean of three independent experiments. 

### 2.14. Sphere-Forming Frequency (SFF) Assay 

To determine the self-renewal nature of the cancer stem cell fraction, we performed the sphere-forming frequency assay of the sorted SCCs and FCCs and the unsorted tumor cells when exposed to TMZ (0–5 mM). To achieve this, we added a single-cell suspension of 1000 cells to each well of the 96-well plate containing the TMZ dosage and incubated them for 4 days. The cells were then fixed and permeabilized, and the nuclei were stained using a solution with a final concentration of 2% PFA (Thermo Scientific; Waltham, MA, USA), 0.01% Triton^TM^ (Roche; Basel, Switzerland) in PBS, and 0.1% DAPI (Thermo Scientific; Waltham, MA, USA). The Gen5 Image software on the Cytation^TM^ 3 Cell Imaging Multi-Mode Reader was utilized to quantify spheres that exhibited a circularity exceeding 0.15 and measured between 50 and 500 µm in diameter.

### 2.15. Bulk RNA-Seq Sample Preparation and Analysis

RNA extraction, RNA-Seq library generation, and sequencing were conducted following previously established protocol [20]. In summary, total RNA was extracted from cells cultured under adherent or serum-free gliomasphere assay conditions and from brain tumor tissue samples using Qiagen kits (Toronto, ON, Canada). Prior to library construction, rigorous quality control steps were implemented to ensure RNA purity and integrity. This involved an initial assessment of RNA purity using Nanodrop2000 (Thermo Scientific; Waltham, MA, USA), agarose gel electrophoresis for RNA integrity and potential contamination, and reconfirmation of RNA integrity using the Agilent 2100 Bioanalyzer (Santa Clara, CA, USA). Subsequently, mRNA was purified and randomly fragmented to initiate cDNA synthesis. For library construction, cDNA fragments of 150–200 bp length were purified to a concentration of 1.5 ng/μL using AMPure XP beads (Beckman Coulter, Beverly, MA, USA). Assessments of library effective concentration and RNA quality were performed using Agilent 2100 Bioanalyzer and Qubit2.0 (Thermo Scientific; Waltham, MA, USA). Finally, the libraries underwent sequencing on the HiSeq platform (Illumina, San Diego, CA, USA) following the manufacturer’s protocols. RNA sequencing data from tumors generated by GL261 cells and CT-2A cells were obtained using the GEO accession numbers GSE107423 [37] and GSE215123, respectively.

The assessment of gene signature enrichment was carried out using GSEA (http://www.broadinstitute.org/gsea/index.jsp, accessed on 16 January 2024). The stem cell signatures utilized in this study were derived from Wong et al. [38] and Harris et al. [39]. The SCC signature was previously described by Hoang-Minh et al. and Yang et al. [19,20]. The GO terms used for the cell migration, cell motility, response to lipid, and lipid catabolism signatures were GO:0016477, GO:0048870, GO:0071396, and GO:0016042, respectively. The KEGG pathway mmu04512 was used as ECM receptor signature. Nominal *p* value < 0.05 and FDR < 0.25 were applied to detect gene set enrichment across different groups. Multivariate principal component analysis (PCA) was utilized to differentiate between tumors derived from SCC or FCC and to compare them with control brain tissues, examining the transcriptomic diversity by using FactoMineR 2.11. Differential expressed genes (DEGs) were extracted by using R limma 3.60.2 (logFC > 1.5 or <−1.5 and BH adjusted *p* value < 0.01) between tumors derived from KR158, GL261, and CT-2A, and their hierarchical clustering analysis was performed by using R pheatmap 1.0.12. 

Data presented in this paper are available from the BioProject database (PRJNA1068559).

### 2.16. Single-Cell RNA Sequencing and Quality Control

CD45-negative cells from KR158 intracranial tumors were isolated and processed for scRNA sequencing as described by Trivedi et al. [40]. In brief, CD45 negative cells were obtained as the flow-through fraction after isolating CD45 positive cells using bead-based selection. A cDNA sequencing library was then prepared using the Chromium Next GEM Single Cell 3′ Reagent Kits v3.1 (Dual Index, 10x Genomics) and sequenced on an Illumina Novaseq 6000. For downstream analysis, Trivedi et al. only used samples with a cell multiplexing oligo assignment probability above 70%, a multiplet rate below 50%, and retained genes that were expressed in at least three cells. Cells with more than 250 genes, 500 UMIs, a complexity above 0.8 (log10 gene count/log10 UMI count), and less than 5% mitochondrial genes were selected using Seurat version 4.045.

### 2.17. Single-Cell RNA-Seq Data Analysis

We utilized SingleR, integrated with the Immgen dataset, to identify cell types [41] from the scRNAseq dataset obtained. Seurat 4.0 was employed to identify differentially expressed genes (DEGs) within cell clusters for subsequent cell type classification. A total of 1000 CD45 negative cells were included in the analysis. We visualized cell populations using the UMAP algorithm via Seurat 4.0. SCCs and FCC were defined using the gene signature and score as previously described [19,20]. The stemness geneset [38] and cell motility (GO:0048870) scores in SCCs were determined by using the Escape package. UMAP projections of scores were generated using the FeaturePlot function in Seurat 4.0.

### 2.18. Statistical Analyses

The results are expressed as mean values ± SEM, with statistical analyses performed using GraphPad Prism 6.0 (GraphPad 10 Software). Statistical tests are indicated in the text. Group comparisons involved either a one-way ANOVA or Student’s *t*-test with 95% confidence intervals. ANOVA-significant groups underwent Tukey’s post hoc analysis. Overall survival was analyzed and compared using log-rank analyses. The flow cytometry results were analyzed using FlowJo 10.10.0 (BD Life Sciences; Franklin Lakes, NJ, USA). 

## 3. Results

### 3.1. Transcriptomic Difference between KR158, GL261, and CT-2A, with KR158 Overexpressing GBM-like Signatures 

**Figure 1:**
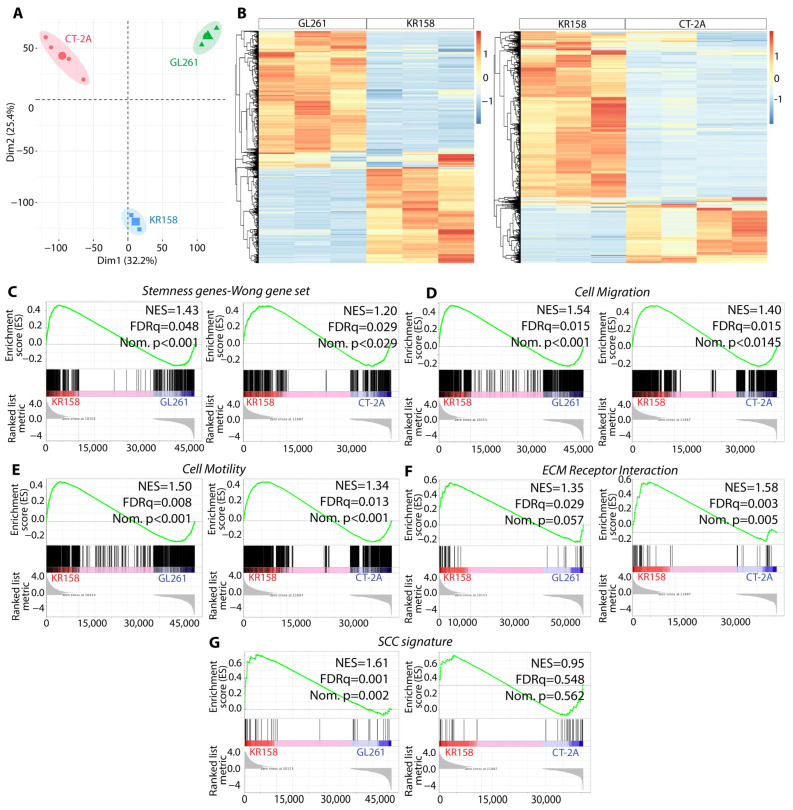
Differential transcriptomic profiles of KR158, GL261, and CT-2A, highlighting upregulation of genes associated with GBM-like features, especially in KR158. (**A**) PCA score plot from bulk RNA sequencing performed on tumors derived from GL261, CT-2A, and KR158 brain tissue (n = 3–4) showed that these tumor models exhibit different transcriptomic profiles. (**B**) Heatmaps showing DEGs of tumors derived from GL261, CT-2A, and KR158 brain tissue. Red and blue indicate relative over- or under-expression of genes, respectively. (**C**–**G**) GSEA between tumors derived from the 3 tumor cell types for the following genesets (n = 3–4 per group); (**C**) stemness (signature from Wong et al. [38]); (**D**) cell migration (GO:0016477); (**E**) cell motility (GO:0048870); (**F**) ECM receptor interaction (KEGG mmu04512); (**G**) SCC gene signatures [19,20]. FDR, false discovery rate; NES, normalized enrichment score; Nom., nominal.

GL261 is one of the most predominantly used preclinical models for high-grade glioma [42,43,44]. However, allograft tumors generated by this model exhibit features including low clonotypic diversity and high antigenicity, contrasting with human GBM [45]. CT-2A, a chemically induced model generated after intracerebral injection of 20-methylcholanthrene, also represents a commonly used syngeneic model of astrocytoma [28,29,30]. The murine model KR158 is another widely used immunocompetent model of glioma, which exhibits several properties that mirror those observed in human GBM [46]. In our effort to assess and compare the capacity of the three models to replicate key characteristics resembling GBM, we first conducted a multivariate principal component analysis (PCA) on tumors originating from KR158, GL261, and CT-2A cells. Our analysis revealed transcriptomic differences between these distinct tumor cell populations (Figure 1A).

The heatmap representations of hierarchical clustering analysis on the DEGs further underscored the transcriptomic distinctions among these tumor models (Figure 1B). Given the significance of stemness and migration in human GBM [47,48,49,50,51,52], we compared the expression levels of various genesets associated with these properties in tumors originating from the three tumor cell types. Study using geneset enrichment analysis (GSEA) on bulk RNA sequencing data indicated elevated expression of stemness and cell migration-related genes in tumors derived from KR158 compared to those from GL261 and CT-2A (Figure 1C–F, Tables S1–S8). Given our goal to investigate a specific subset of cells, namely, SCCs, in an immunocompetent model of GBM, it was critical to examine the transcriptomic profile of these models in relation to genes previously associated with such cells or cellular states in human GBM [19,20]. Interestingly, our findings revealed an upregulation of the SCC gene signature in tumors derived from KR158 as compared to those from GL261; however, no difference was noted between KR158 and CT-2A cells (Figure 1G, Tables S9 and S10). This observation suggests that the KR158 model may be more relevant when studying GBM stem cells, including SCCs.

### 3.2. scRNA Sequencing Revealed Cellular Diversity in KR158 Tumors, Exhibiting Similarities with the Heterogeneity Observed in hGBM

**Figure 2:**
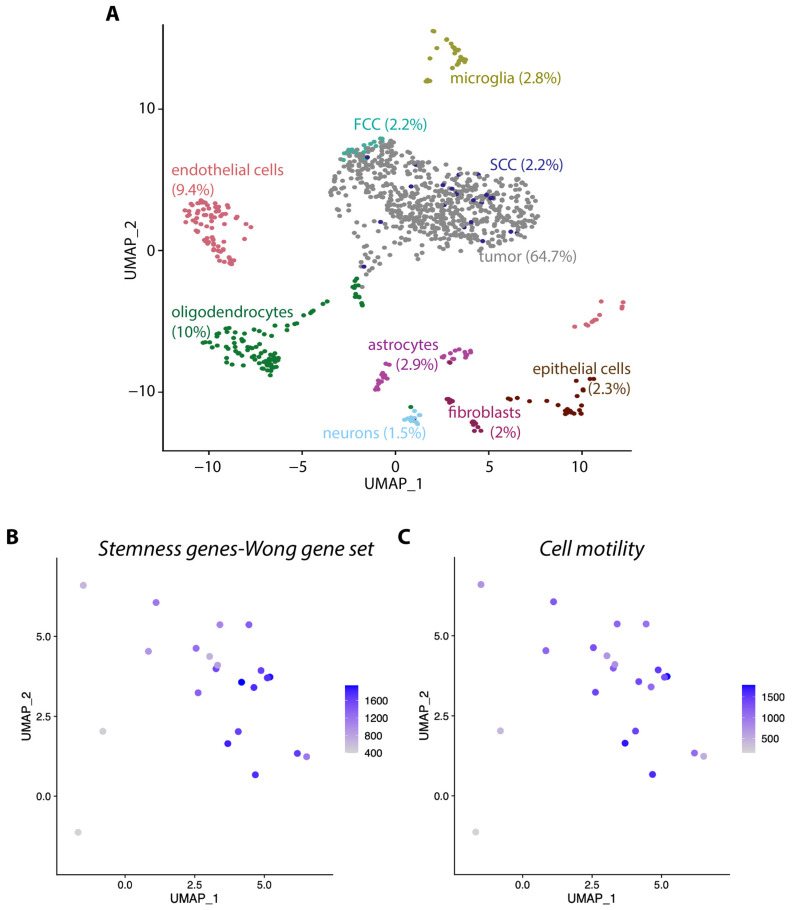
scRNA sequencing revealing cellular diversity in KR158 tumors. (**A**) UMAP visualization of pooled scRNA-seq data of 1000 CD45 negative cells of the tumor microenvironment from tumors derived from KR158 brain tissue. We identified 8 clusters, including tumor cells, microglia, endothelial cells, oligodendrocytes, astrocytes, fibroblasts, neurons, and epithelial cells. Tumor cell fraction contains subpopulation of SCCs (green) (22 cells) and FCCs (red) (22 cells). The respective percentage of each cell type is indicated in parenthesis. (**B**,**C**) Stemness geneset [38] (**B**) and cell motility (GO:0048870) scores (**C**) of each single cell were determined by using the Escape package. Scores were protected to UMAP by using the Seurat 4.0 FeaturePlot function.

Utilizing a single-cell RNA (scRNA) sequencing dataset obtained from KR158 intracranial tumors [40], we analyzed gene expression in a thousand excised cells. These cells were classified into eight distinct cellular clusters, each representing a major cell type, including tumor cells, microglia, endothelial cells, oligodendrocytes, astrocytes, fibroblasts, neurons, and epithelial cells (Figure 2A). 

This result illustrates the cellular heterogeneity within the tumor microenvironment of this model, a characteristic also identified in human disease. The tumor cell group was subdivided into two distinct subpopulations, namely, the SCC and FCC cellular clusters, distinguished by the expression of specific gene signatures, as previously described in hGBM [20]. Notably, the SCC population displayed significant heterogeneity, evident in its widespread distribution on the UMAP plot, contrasting with the more compact cluster formed by the FCC cells (Figure 2A). Additionally, in alignment with our observations in hGBM, the SCCs demonstrate elevated expression of genes associated with stemness and cellular motility, which are key features of hGBM (Figure 2B,C) [53,54]. Collectively, these results support the significance of the KR158 model for unraveling the tumor microenvironment and exploring the characteristics of SCCs.

### 3.3. KR158 Cells Expanded Using the Gliomasphere Assay Generate Glioblastoma-like Tumors

**Figure 3:**
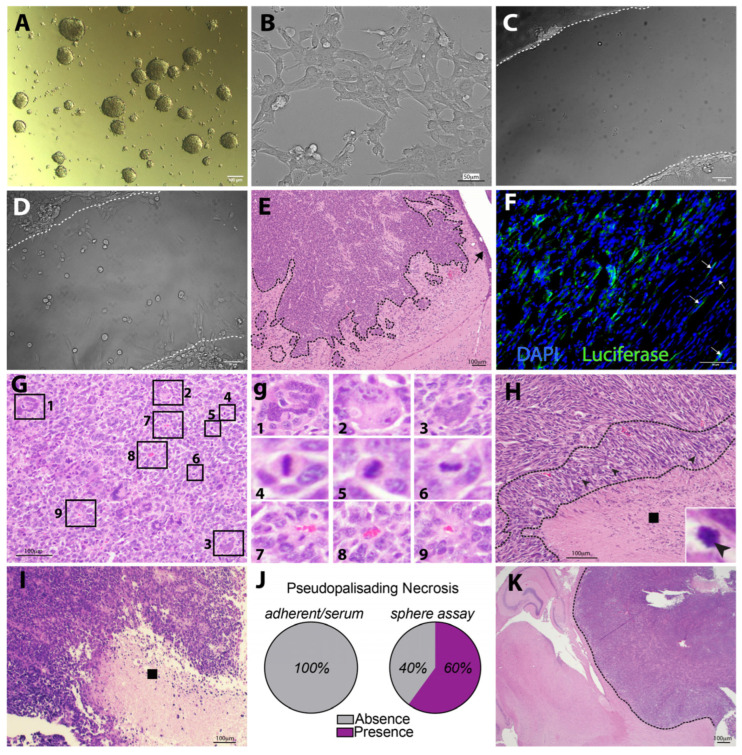
KR158 murine glioma cells grown in the glioma sphere assay demonstrate tumor formation characterized by hallmarks of high-grade glioma. (**A**) Brightfield image of Luciferase-expressing KR158 cells (KLuc) murine glioma cells grow as spheres when cultured in serum-free medium supplemented with epidermal growth factor (EGF) and basic human fibroblast growth factor (hFGF). (**B**) Brightfield image of KLuc cells cultured in adherent and serum-containing conditions. (**C**,**D**) Scratch-wound assay; brightfield images acquired at time 0 (**C**) and 23 h (**D**) show the migratory behavior of the cells that were expanded in the gliomasphere assay. (**E**) Hematoxylin and eosin staining (H&E) show that intracranial implantation with KLuc cells cultured in serum-free conditions in C57BL6 mice demonstrate tumorigenicity with ability to generate tumors exhibiting GBM characteristics including infiltration. Tumor leading edge denoted by the dotted line, → indicates subpial spreading. (**F**) Luciferase labeling (green) further confirms invasive properties of KLuc into the host brain parenchyma. White arrowheads indicate luciferase^+^ cells that have migrated away from the tumor core, infiltrating the surrounding brain parenchyma. Nuclei are labeled with DAPI (blue). (**G**) H&E staining of tumors developed from cells cultured in the gliomasphere assay depicts the presence of giant cells, identified as #1–3, mitotic figures (#4–6), and clustering in perivascular regions (#7–9). Panel (**g**) represents higher magnification insets indicated by the rectangles in panel (**G**). (**H**) Presence of pseudopalisading necrosis further validates the formation of high-grade glioma-like disease from KLuc cells expanded in gliomasphere serum-free medium. ◾ indicates necrotic region, dotted line indicates pseudopalisade, ➤ indicates pyknotic nuclei. (**I**) H&E labeling of brain sections of animals implanted with KLuc cells cultured in serum-containing conditions. Image presenting a necrotic area lacking the GBM characteristic of pseudopalisading. (**J**) Fraction of necrotic regions displaying pseudopalisading. (**K**) Image indicating the limited infiltration of tumors generated by cells cultured in adherent serum-containing conditions. Tumor leading edge denoted by the dotted line. Shown are representative images from 5 mice per group.

With the goal of supporting the cancer stem cell phenotype in an immunocompetent model of GBM, KR158 cells were cultured using the serum-free gliomasphere assay [25,27]. Under these conditions, KR158 cells formed spheres (Figure 3A), as opposed to an adherent monolayer growth pattern when cultured in standard conditions containing serum (Figure 3B). 

The scratch-wound assay demonstrated that cells expanded under the defined gliomasphere conditions maintain their migration capacity, a critical characteristic of GBM cells (Figure 3C,D, Supplementary Video S1). Importantly, the orthotopic transplantation of a single-cell suspension of luciferase-expressing KR158 cells (KLuc) expanded in the gliomasphere assay, leading to the development of brain tumors. These tumors exhibited hallmark features of grade 4 glioma, such as extensive invasion with notable subpial spread (Figure 3E,F). Cellular and nuclear pleomorphism with giant cells were also observed, along with abundant mitosis and perivascular aggregates (Figure 3G,g). The presence of pseudopalisading necrotic regions characterized by a hypercellular nuclei rearrangement surrounding irregular foci of tumor necrosis containing pyknotic nuclei was also observed (Figure 3H, Supplementary Figure S1A). Notably, tumors originating from cells grown in monolayer serum-containing conditions also exhibited evidence of necrosis, albeit much less frequently and without well-defined palisades and a sparser cellular arrangement surrounding the necrotic foci (Figure 3I,J, Supplementary Figure S1B). Comparatively, 60% of the necrotic areas displayed pseudopalisading in the tumors derived from cells cultured in the gliomasphere assay (Figure 3J). Furthermore, tumors derived from serum-containing cultures exhibited a less extensive infiltrative phenotype, displaying a more circumscribed growth pattern (Figure 3K), suggesting a lower grade. These findings highlight the tumorigenic nature of KR158-derived gliomaspheres, generating tumors that exhibit characteristics more akin to grade 4 tumors. This distinction is evident in the presence of high infiltration and well-defined pseudopalisading necrosis, unlike the grade 3-like malignancies lacking such features when cultured under adherent serum-containing conditions.

### 3.4. The Gliomasphere Assay Enhances Stemness Gene Signature in KR158 Cells

**Figure 4:**
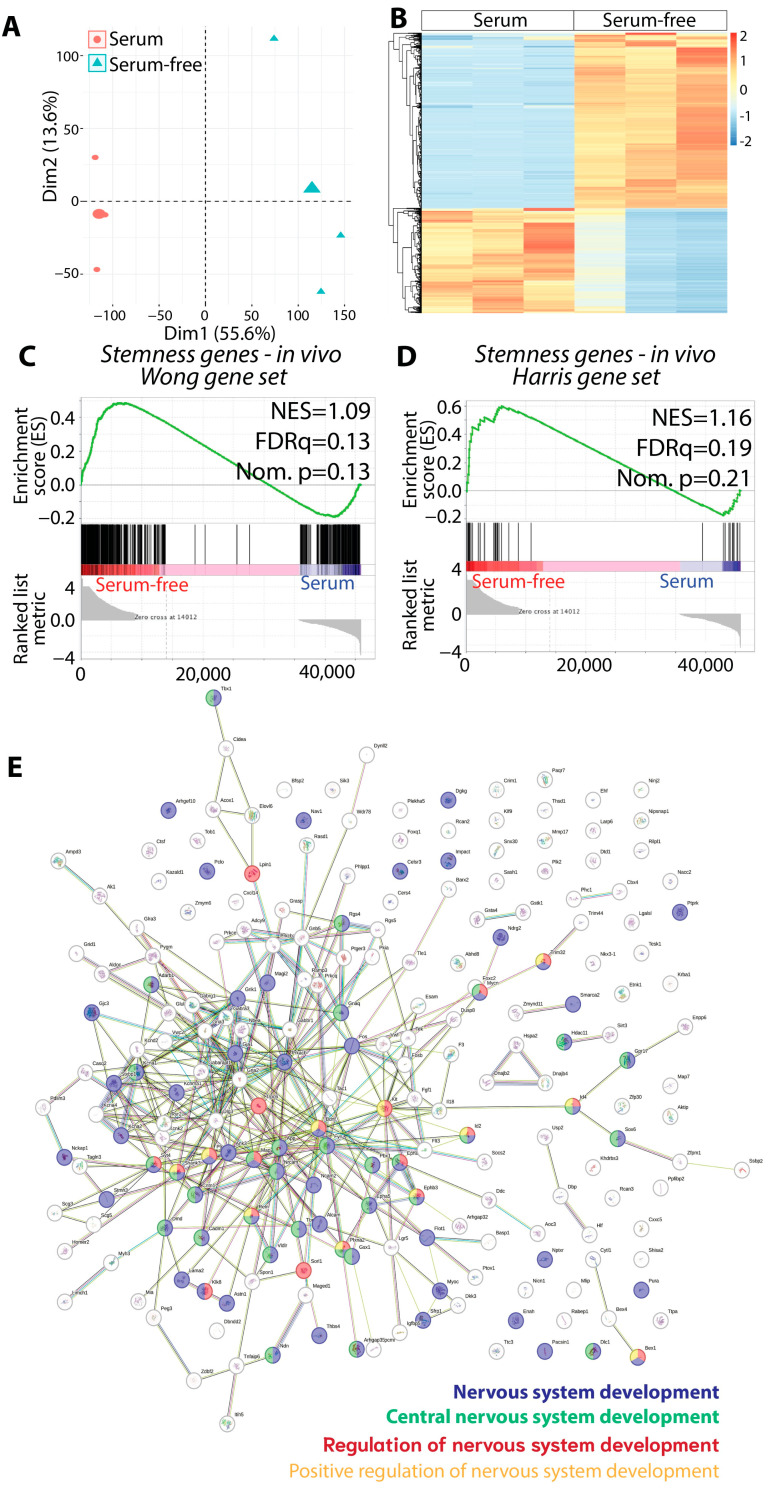
KR158 murine glioma cells grown in serum-free medium are enriched in stemness genes. (**A**) PCA score plot from bulk RNA sequencing performed on brain tumor tissue generated by KR158 cells cultured adherent in serum or serum-free gliomasphere assay (*n* = 3), showed that these tumor models exhibit different transcriptomic profiles. (**B**) Heatmap showing DEGs of tumors formed by KR158 cells cultured in serum or serum-free gliomasphere assay (*n* = 3). Red and blue indicate relative over- or under-expression of genes, respectively. (**C**,**D**) GSEA of RNA sequencing data from in vivo tumors (*n* = 3 per group) shows an enrichment of stemness gene signature in tumors generated by cells cultured in serum-free conditions compared to cells expanded in serum-containing conditions using gene signature from (**C**): Wong et al. [38] and (**D**) Harris Brain Cancer Progenitors gene set [39], systematic name M1694; FDR, false discovery rate; NES, normalized enrichment score; Nom., nominal. (**E**) Search Tool for the Retrieval of Interacting Genes/Proteins (STRING) database analysis identified computational predictions defining functional associations between the genes upregulated in the KR158 cells expanded in the gliomasphere assay and mechanisms regulating the development of the nervous system.

Upon confirming through histopathological evidence that KR158-derived gliomasphere tumors align better with grade 4 tumor characteristics, we compared the transcriptomic profiles of KR158 cells grown in gliomasphere and adherent conditions. Utilizing multivariant PCA from bulk RNA sequencing, we identified notable transcriptomic diversities between these two cell populations (Figure 4A). 

This difference was further characterized by a hierarchical clustering analysis of DEGs, visualized in a heatmap (Figure 4B). GSEA indicated an increased stemness in cells grown in the gliomasphere assay, evidenced by an enriched stem cell gene signature in the tumors generated from these cells when compared to those grown under monolayer serum-containing conditions (Figure 4C,D, Tables S11 and S12) [38,39]. Utilizing the STRING platform (https://string-db.org/, accessed on 17 January 2024) for network analysis, it was observed that the genes showing significant upregulation in cells cultured via the gliomasphere assay, as indicated in Tables S11 and S12, formed a network associated with the regulation of nervous system development (Figure 4E, Table S13). Examples of genes present in this network associated with stem cells and the regulation of their self-renewal and differentiation include Mycn [55,56,57,58,59], Sox6 [60], Ncam2 [61], Fgf1 [62,63,64], Kit [65], and Larp6 [66,67,68]. To investigate whether the observed phenotypic variances between the two assays (serum-free sphere vs. serum-containing monolayer) stemmed from genetic modifications induced by the distinct culture methods, we conducted a short tandem repeat (STR)-based DNA profiling analysis. Through multiplex PCR, we compared the number of alleles repeat motifs across different DNA loci. Remarkably, our analysis revealed a 100% match, based on the 19 different STR markers, between both groups (Supplementary Figure S2). This suggests a lack of minimal genetic drift between the two culture conditions throughout the duration of this study. Consequently, it implies that the observed phenotypic differences may arise from epigenetic regulation rather than genetic alterations.

### 3.5. Evidence of SCCs in KR158 Gliomasphere Cultures Showing Similarities to SCCs in hGBM

**Figure 5:**
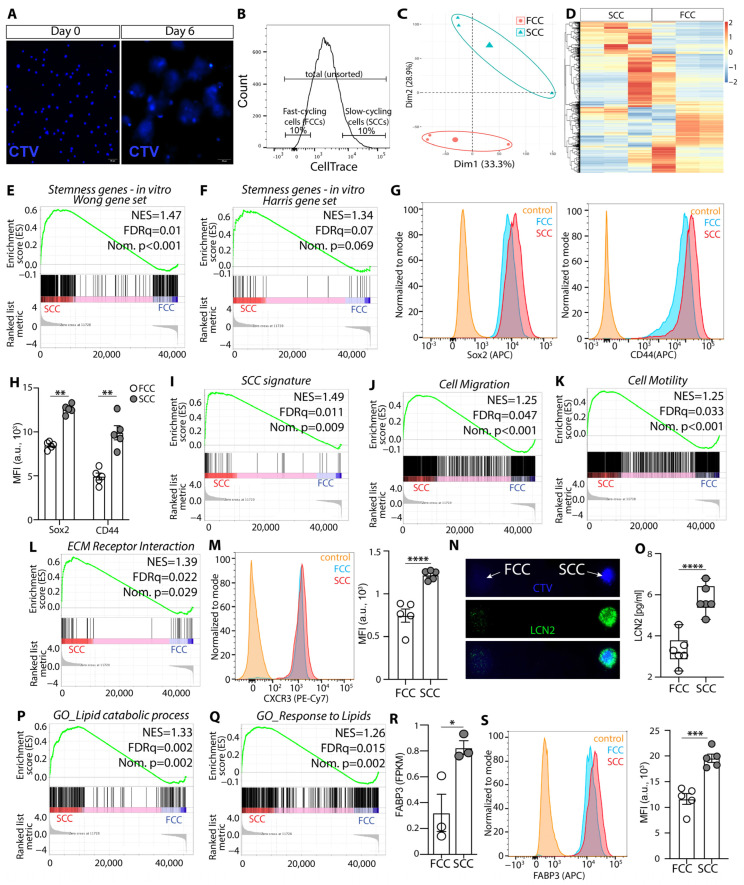
SCC KR158 cells grown in serum-free conditions up-regulate stemness, migration, and lipid metabolism characteristics compared to FCC cells. (**A**) Cell trace labeling of KR158 cells grown in the gliomasphere assay at Day 0 and Day 6. (**B**) Fluorescent-activated cell sorting of KLuc cells were performed on Day 6; top 10% of the cells with highest CTV retention were sorted and defined as SCCs and the bottom 10% of cells with least CTV retention as FCCs. (**C**) PCA score plot from bulk RNA sequencing performed on tumors derived from SCC and FCC brain tissue (*n* = 3), showed that these tumor models exhibit different transcriptomic profiles. (**D**) Heatmap showing DEGs of tumors derived from SCC and FCC brain tissue (*n* = 3). Red and blue indicate relative over- or under-expression of genes, respectively. GSEA of in vitro RNA-Seq datasets between gliomasphere serum-free cultured SCC (*n* = 3) and FCC (*n* = 3); (**E**) stemness geneset [38]; (**F**) stemness geneset (M1694) [39]. (**G**) Flow cytometric analysis (representative histograms) of Sox2 and CD44 expression in SCCs and FCCs. (**H**) Bar diagram quantifying flow analysis for Sox2 and CD44 expression in SCC vs. FCC, n = 5, *t*-test ** *p* < 0.01. (**I**) SCC gene signature [19,20]; (**J**) cell migration (GO:0016477); (**K**) cell motility (GO:0048870); (**L**) ECM receptor interaction (KEGG mmu04512); FDR, false discovery rate; NES, normalized enrichment score; Nom., nominal. (**M**) Representative histogram comparing CXCR3 expression in SCCs and FCCs using flow cytometry, and bar diagram (n = 5), **** *p* < 0.0001, *t*-test. (**N**) Immunofluorescence microscopy images of a representative single SCC (CTV positive, blue) and FCC (CTV negative/low) labeled for LCN2 (green). (**O**) Quantification using ELISA of LCN2 secreted by SCCs and FCCs, *n* = 6, ****, *p* < 0.001, *t*-test. (**P**) Lipid catabolic process (GO:0016042); (**Q**) response to lipid (GO:0071396). (**R**) Bar graph comparing FABP3 gene expression level in SCC and FCC from bulk RNA sequencing (*n* = 3), *, *p* < 0.05, *t*-test. (**S**) Flow cytometric comparison of FABP3 protein level in SCCs and FCCs, *n* = 5, *** *p* < 0.005, *t*-test.

Considering their ability to recapitulate the histological features of human GBM and their heightened stemness, KR158 cells grown in the gliomasphere assay were then used to identify and characterize SCCs in comparison to FCCs. KR158 SCCs were defined as CellTrace dye-retaining cells (Figure 5A,B), as per a previously established method [16,17,19,20]. Bulk RNA sequencing showed differential clustering between the different tumor types through PCA (Figure 5C), which is also visualized through a heatmap representing the hierarchical clustering analysis of differentially expressed genes (Figure 5D). 

Consistent with our findings in human GBM [19], GSEA from RNA sequencing showed that stemness genes were expressed at higher levels in murine glioma SCCs compared to FCCs when cultured in the gliomasphere assay (Figure 5E,F, Tables S14 and S15). Additionally, SCCs exhibited a significant overexpression of stemness markers Sox2 and CD44 compared to FCCs at a protein level (Figure 5G,H). Also, the genes corresponding to SCC signature (Figure 5I, Table S16), cell migration (Figure 5J, Table S17), cell motility (Figure 5K, Table S18), and ECM receptor interaction (Figure 5L, Table S19) were expressed at significantly higher levels in murine glioma SCCs compared to FCCs. Moreover, CXCR3, one of the top enriched genes in the SCCs associated with cell migration and motility, was also observed to be significantly upregulated at a protein level in SCCs (Figure 5M, Tables S17 and S18). LCN2, the most upregulated transcript in the migration signature in SCC, encodes a circulatory protein that plays a crucial role in both migration and fatty acid metabolism [69,70]. LCN2 also demonstrated significant protein overexpression in SCCs compared to FCCs, as evidenced by immuno-cytochemistry and ELISA measuring the quantity of secreted protein (Figure 5N,O). Additionally, we found that lipid catabolic processes and responses to lipid-related genes were also upregulated in SCCs, supporting a distinct metabolic profile similar to what we previously described in human GBM cells (Figure 5P,Q, Tables S20 and S21) [19]. While FABP3 was not included in the metabolic signatures mentioned above, we assessed the expression level of this chaperone protein, which we demonstrated regulates lipid trafficking in glioblastoma and is enriched in human GBM patient-derived SCC [19]. Our findings indicate that similar to their human counterparts, KR158 SCCs also exhibit higher levels of FABP3 transcript and protein (Figure 5R,S).

### 3.6. Murine KR158 SCCs Form GBM-like Tumor with Heightened Stemness and Aggressive Phenotype

**Figure 6:**
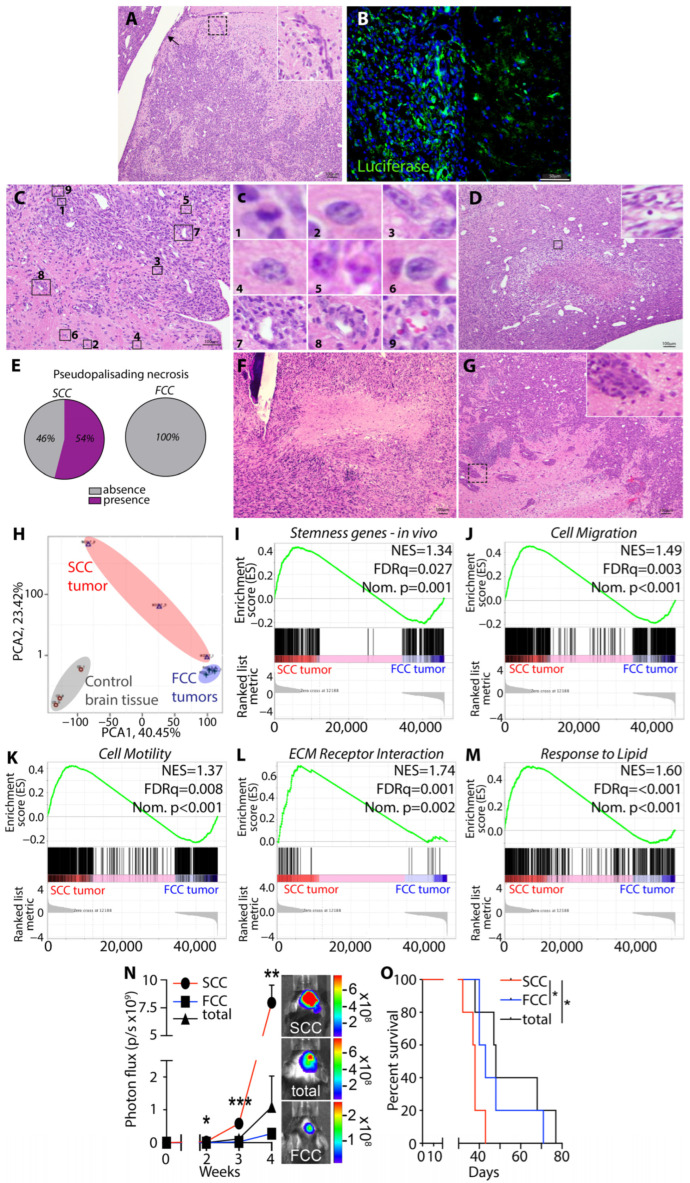
Murine glioma SCCs grown in the gliomasphere assay are tumorigenic with enhanced stemness. SCCs demonstrated the ability to generate high-grade glioma-like disease as seen by high levels of tumor cell infiltration observed by H&E ((**A**), → indicates subpial spreading). The inset represents a higher magnification of the invasive front exhibiting tentacle-like infiltration. (**B**) Luciferase labeling further illustrates the infiltrative nature of the cells. Nuclei are labeled with DAPI (blue). (**C**) Presence of mitotic figures (#1–6) and perivascular clustering (#7–9). Panel (**c**) represents higher magnification insets of the panel (**C**) rectangles. (**D**) Pseudopalisading necrosis in SCC tumors (inset showing a higher magnification of a pyknotic nucleus). (**E**) Pie charts indicating the percentage of necrotic areas exhibiting pseudopalisading. (**F**) H&E staining of FCC tumors illustrating the presence of necrosis lacking pseudopalisading. (**G**) FCC tumors show features of infiltration. The inset represents a higher magnification of the invasive front showing grouped infiltration. (**H**) PCA score plot from RNA sequencing performed on tumors derived from SCCs and FCCs and control brain tissue (*n* = 3) showed that these different tumor cell populations maintain transcriptome diversity upon tumor progression. GSEA of in vivo RNA-Seq datasets between tumors derived from SCCs and FCCs (*n* = 3 per group) comparing the following signatures (**I**) stemness [38]; (**J**) cell migration (GO:0016477), (**K**) cell motility (GO:0048870); (**L**) ECM receptor interaction (KEGG mmu04512) and (**M**) response to lipids (GO:0071396). FDR, false discovery rate; NES, normalized enrichment score; Nom., nominal. (**N**) Intracranial tumor growth monitored using bioluminescence in vivo imaging capturing luciferase activity in immunocompetent mice implanted with SCCs and FCCs (*n* = 5). *t*-test, *, **, ***, *p* < 0.05, *p* < 0.01, *p* < 0.005, respectively. (**O**) Kaplan–Meier survival curves of immunocompetent animals implanted with SCCs compared to FCCs or total unsorted cells. * *p* < 0.05, log-rank test.

The positive tumorigenicity of KR158 SCCs was evident through their intracranial implantation immediately after FACS isolation, resulting in the formation of brain tumors displaying hallmark histopathological features of GBM. These characteristics included infiltration and subpial spreading (Figure 6A,B). 

Additionally, high levels of mitotic activity were evident, illustrated by cells in metaphase, anaphase, and telophase (Figure 6C,c1–3). The presence of multipolar atypical mitotic figures was also noted (Figure 6C,c4–6). Furthermore, the presence of red blood cells and perivascular aggregation was demonstrated (Figure 6C,c7–9), along with the presence of pyknotic nuclei and pseudopalisading necrosis observed in about 55% of the necrotic foci (Figure 6D,E, Supplementary Figure S3). In contrast, while tumors derived from FCCs also exhibited necrosis, well-defined pseudopalisading was not evident (Figure 6E,F, Supplementary Figure S4), and their pattern of infiltration appeared less dispersed (Figure 6G). The differentiation between these tumors was further illustrated by bulk RNA sequencing, revealing a distinct clustering pattern through PCA (Figure 6H). Notably, the FCC cluster appeared more compact in comparison to the more dispersed SCC fraction, consistent with the observations from scRNA sequencing (Figure 2A). This pattern implies a higher degree of cellular diversity within the SCC lineage compared to the FCC lineage. GSEA indicated an enrichment of stemness genes in SCC-derived tumors (Figure 6I, Table S22). The SCC tumors also displayed higher transcriptional expression of genes associated with cell migration, cell motility, and ECM receptor interaction (Figure 6J–L, Tables S23–S25). Also, the SCC-derived tumors were found to be enriched with genes regulating lipid metabolism (Figure 6M, Table S26). Crucially, from a functional perspective, these SCC-derived tumors were linked to a more aggressive phenotype. This was evidenced through the longitudinal monitoring of tumor volumes, measured using an IVIS Spectrum imaging system that captures bioluminescence associated with luciferase activity. Tumors generated by the intracranial transplant of luciferase-expressing SCCs showed faster progression compared to the other groups (Figure 6N), resulting in a shorter survival time (Figure 6O). 

### 3.7. Murine KR158 SCCs Are More Tolerant to TMZ Compared to FCCs

**Figure 7:**
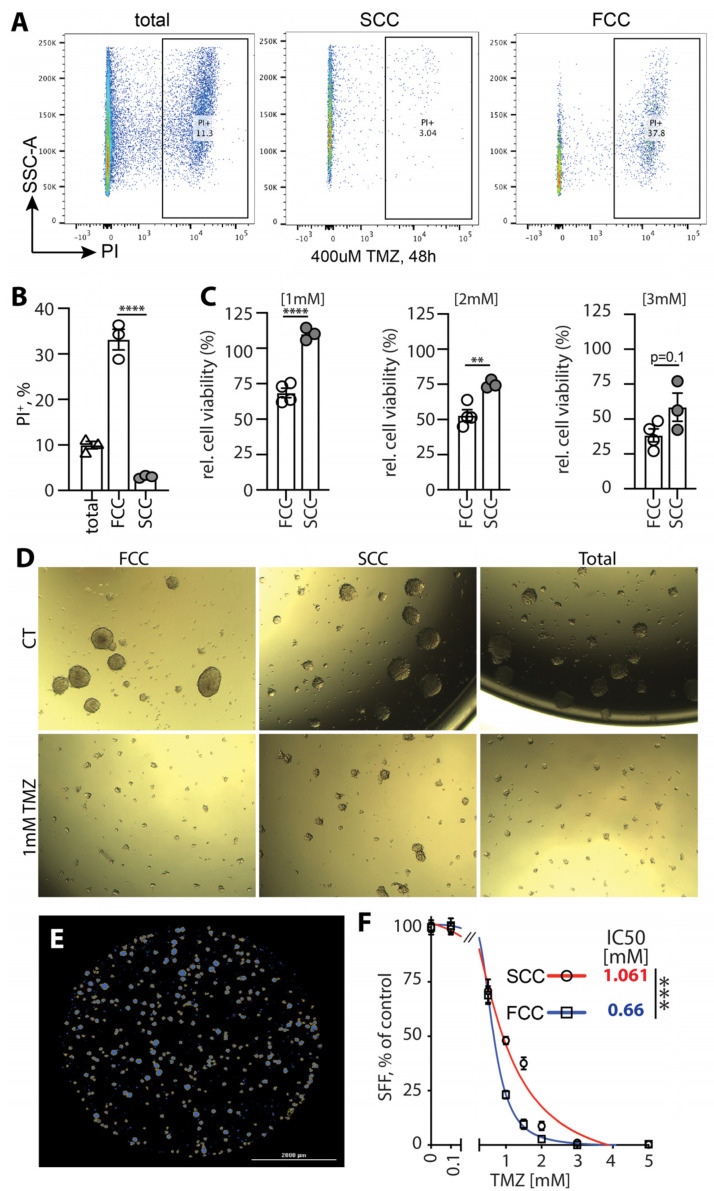
SCC KR158 cells are more resistant to TMZ. (**A**,**B**) PI incorporation assay within the tumor cells upon 48 h of treatment with 400µM TMZ; Representative flow dot plots (**A**) with quantification of three independent experiments, (**B**) **** *p* < 0.001, one-way ANOVA with Tukey post-test)**.** (**C**) Representative bar diagram of relative cell viability of SCCs and FCCs using the fluorescence-based CyQUANT cell proliferation assay performed 48 h after exposure to 1, 2, and 3 mM TMZ. Values represent mean +/− SEM, **** *p* < 0.001, ** *p* < 0.01, *t*-test. Brightfield (**D**) and fluorescent (DAPI) (**E**) examples of gliomaspheres generated from the different KR158 cell populations treated with TMZ. (**F**) TMZ dose-response was evaluated by comparing the SFF between SCCs and FCCs treated with a range of concentrations (0.1, 0.5, 1, 1.5, 2, 3, 5 mM). Values represent mean +/− SEM, expressed as percentage of untreated conditions, *n* = 10–20. IC50s for each population were calculated using non-linear regression sigmoidal dose-response, *** *p* < 0.005.

Based on the aforementioned phenotype of SCCs and the tumors they generate, we hypothesized that these cells may be less sensitive to chemotherapy. To evaluate the impact of the chemotherapeutic agent TMZ on the viability of freshly sorted SCCs, FCCs, and total unsorted tumor cells, we performed an in vitro PI incorporation assay. Similar to human GBM, murine SCCs displayed lower sensitivity to TMZ than the other cell populations (Figure 7A,B). 

The fluorescent-based CyQuant assay was also used to compare cell numbers in the TMZ-treated cultures, revealing that SCCs had enhanced viability compared to FCCs (Figure 7C). Additionally, the sphere-forming frequency (SFF) assay indicated that SCCs exhibited significantly higher self-renewal activity in response to TMZ treatment than FCCs (Figure 7D–F), with an IC50 of 1.061 mM versus 0.66 mM for FCCs, further confirming their greater tolerance to the chemotherapeutic agent. 

Together, these findings also support the conclusion that the KR158 cells adapted to the gliomasphere assay contain SCCs with properties and functions, including sphere-forming ability, positive tumorigenicity, characteristic metabolic gene profile, and enhanced stemness and treatment resistance, similar to those described in human GBM [16,19,20]. This study validates our immunocompetent murine model for investigating the relationship between tumor-immune infiltrates and GBM cells, specifically SCCs.

## 4. Discussion

The intricate heterogeneity within GBM underscores its complexity and poses challenges in therapeutic approaches. The emergent understanding of CSCs, particularly slow-cycling cancer stem cells, supports their significant contribution to this heterogeneity and treatment resistance in GBM. Moreover, while the metabolic diversity within tumor cells that shapes their unique metabolic microenvironments has been identified [1,19], the precise influence of this heterogeneity on the immune landscape and disease progression remains largely elusive [5]. This study is aimed to delineate and characterize GSCs, particularly SCCs, in an immunocompetent murine model of GBM. Our goal was to demonstrate that these cells, isolated from a specific murine model of GBM, mimic key phenotypic and functional traits observed in their human counterparts. Validating this paradigm in murine models will support their use in exploring the interactions between SCCs and the immune system, encompassing the study of their metabolic communications and dependencies. This deeper understanding of how tumor cell metabolic properties and diversity impact the tumor-immune microenvironment will guide therapeutic strategies toward modulating this metabolic interplay.

GBM models played a crucial role in understanding tumor dynamics. Patient-derived models, patient-derived xenografts, murine models like syngeneic models, and genetically engineered mouse models (GEMMs) offer distinct advantages and limitations in investigating the role of GSCs within the TME, especially the tumor-immune microenvironment. These diverse tools vary in their ability to mimic the human tumor microenvironment. Patient-derived in vitro models, including primary cell cultures and tumor organoids, offer relatively easy accessibility for experimental manipulations and high-throughput drug screening, molecular profiling, and mechanistic studies. However, these models may lack key aspects of the tumor heterogeneity and microenvironment complexity compared to patient-derived xenografts (PDXs), which maintain the molecular, histological, and architectural characteristics of the original tumor, thus closely mirroring the genetic landscape of the patient tumor and thus maintaining their clinical relevance. Although clinically relevant, PDXs require an immunocompromised system to prevent graft rejection, limiting the investigation of the role of the immune system in tumor progression and treatment response.

Conversely, syngeneic murine models can offer insights into tumor-stroma interactions, especially tumor–immune cell communication. However, recapitulation of the diffuse and infiltrative nature of GBM has been challenging to achieve in murine models. Therefore, presenting this unique property of GBM in mice is desirable to more accurately model tumor–stroma interactions and GBM cell behavior. Examples of murine models of GBM include GEMMs in which tissue specific promoters are used for oncogenic transformation in neural stem and progenitor cells such as nestin-, GFAP-, CNPase-, and S100beta-positive cells [71,72,73,74,75,76,77,78,79]. Other syngeneic models formed through various induction methods can also include GL261, CT-2A, and KR158 [28,29,80,81,82,83,84,85,86,87]. Together, these murine models provide a molecular insight into the impact of genetic mutations on disease initiation, progression, and treatment outcome. However, being more homogenetic in nature, they are less reflective of the intratumoral genomic and phenotypic heterogeneity of GBM TME [88]. Another limitation is the type of architecture of the tumors they generate, with limited tumor infiltration of the brain parenchyma and more circumscribed edges, contrasting with the highly invasive architecture typically observed in GBM patients [16,89]. Additionally, these models, notably GL261 and CT-2A, exhibit marked immunogenicity with elevated MHC I expression, differing from the immunological profile observed in human pathology [45,90,91,92,93]. In contrast, the KR158 model displays lower immunogenicity and demonstrates inherent resistance to checkpoint inhibition, mirroring characteristics found in human GBM [46]. This particular trait could potentially render this model more relevant, especially in the evaluation of experimental immunotherapies. With the goal of overcoming some of these limitations, especially in recapitulating tumor heterogeneity and the morphological characteristics of human GBM, including infiltration into the surrounding immunocompetent brain parenchyma, we adapted the KR158 cell murine model to the gliomasphere assay. This assay was developed to culture, enrich, and study GSCs, which form tumors recapitulating the key architectural characteristics of GBM, such as infiltration [26,27,94,95]. This assay was designed to retain tumor heterogeneity, including the presence of GSCs alongside their differentiated progenies, facilitating the study of tumor cell hierarchy and diversity, and performing functional assays, such as investigating self-renewal capacity, differentiation potential, drug response, and tumorigenicity. Gliomasphere cultures maintain the phenotypic and genetic characteristics of the original tumor [4,27] while also preserving stem cell-like properties. These conditions also conserve the self-renewal and differentiation abilities of GSCs, contributing to the diversity within the tumor. 

Studies have shown that murine glioma cell lines, such as GL261 and CT-2A, when cultured as gliomaspheres (GL261-NS; CT-2A-NS), demonstrate an enrichment for cancer stem-like cells. This enrichment is notably greater compared to cells expanded under serum-containing monolayer-adherent conditions. A histopathological examination of tumors generated from GL261-NS and CT-2A-NS revealed some characteristics consistent with high-grade glioma, presenting an aggressive infiltrative phenotype within the murine brain parenchyma [29,96]. Specifically, CT-2A-NS tumors manifest both distant and adjacent satellite lesions but lack the formation of pseudopalisading necrosis. However, our study demonstrated that culturing KR158 murine cells in the gliomasphere assay enriched cells with greater stemness, compared to serum-containing adherent conditions and GL261 and CT-2A. Additionally, KR158 cells expanded as gliomaspheres generated tumors exhibiting all the key morphological features of GBM, including a high level of infiltration and well-defined pseudopalisading necrotic areas. The malignant lesions reveal chromosomal heterogeneity, including the presence of atypical mitotic features, especially multipolar mitotic figures. This phenomenon has been previously documented in GBM tissues and is known to significantly contribute to the induction of aneuploidy in glioblastoma cells. These findings further underscore the significance of the use of this model in understanding the disease [97]. 

Importantly, we also demonstrated the existence of SCCs in these cultures, which showed upregulated stemness programs, positive tumorigenicity, and greater resistance to treatment, which are properties also defined in GBM patient-derived SCCs [16,19,20]. Even though our study focused on the characterization of KR158 cells with respect to modeling CSCs and SCCs due to the notable expression of SCC genes in CT-2A, as well (Figure 1G), we also demonstrated that the presence of SCCs in CT-2A cultures expanded in the gliomasphere assay (Supplementary Figure S5).

Our previous investigations indicated in hGBM that SCC and FCC populations represent independent lineages with limited phenotypic and functional overlap, contributing to the heterogeneous landscape of GBM [20]. The current study also supports such a hypothesis, as tumors originating from SCC or FCC and composed by their respective progenies demonstrate significantly different transcriptomes (Figure 6H), treatment responses (Figure 7), and disease projections (Figure 6A–G,N,O). Although both cellular fractions displayed invasive characteristics, their infiltration patterns appeared distinct. The SCC group exhibited highly dispersed and individually penetrating cells, while the FCC tumors showed a more collective yet diffused invasion with the formation of satellite lesions nearby in the TME. Of note, this observation is qualitative rather than quantitative, supporting the need for further investigation to elucidate the differences in diffusion patterns and the underlying mechanisms regulating these properties. Furthermore, the considerable gene expression dispersion among SCC tumors, contrasting with the clustered expression pattern observed in FCC tumors in the UMPA and PCA plot (Figure 2A and Figure 6H), could suggest increased heterogeneity and plasticity in the SCC lineage. This notion is reinforced by the enriched stemness signature evident in SCCs (Figure 5I and Figure 6I). The distinctions observed between SCCs and FCCs not only could be attributed to inherent lineage differences but also to phenotypic plasticity regulated by environmental factors, including the immune microenvironment. However, the precise vertical and horizontal hierarchical relationship between SCCs and FCCs has yet to be definitively determined, potentially requiring experiments involving lineage tracing followed by comprehensive functional and phenotypic analyses conducted side by side.

## 5. Conclusions

KR158 cells expanded in the gliomasphere assay in serum-free conditions replicate in vivo the diversity and heterogeneity of a GBM-like tumor microenvironment in the context of a maintained immune contexture. Additionally, the infiltrative phenotype of the model, combined with the existence of the slow-cycling cell paradigm, further supports its significance in investigating the involvement of GSCs and SCCs in GBM pathology and deciphering the complexity of the TME. Specifically, this model will provide an important platform to investigate the specific connections between SCCs and the immune system and assess how these communications govern disease presentation, progression, and resistance to treatment. Importantly, this model presents an opportunity to evaluate strategies that target the interplay between SCCs and immune cells and assess their potential therapeutic effects. 

## Data Availability

Data supporting the findings within this study are presented within the article and are available from the corresponding author upon reasonable request.

## Supplementary Materials

The following supporting information can be downloaded at https://www.mdpi.com/article/10.3390/cells13110938/s1: Supplemental Video S1. Scratch-wound assay. Time-lapse imaging shows the migration capacity of KR158 cells expanded in the gliomasphere assay during multiple passages prior to conducting the scratch assay. The video comprises 283 frames captured every 10 min and presented at a rate of 5 frames per second. Table S1. Comparison of GSEA output: stemness geneset [38] expression levels in tumors derived from KR158 cells cultured with serum versus GL261 cells. Table S2. Comparison of GSEA output: stemness geneset [38] expression levels in tumors derived from KR158 cells cultured with serum versus CT-2A cells. Table S3. Comparison of GSEA output: migration geneset (GO:0016477) expression levels in tumors derived from KR158 cells cultured with serum versus GL261 cells. Table S4. Comparison of GSEA output: migration geneset (GO:0016477) expression levels in tumors derived from KR158 cells cultured with serum versus CT-2A cells. Table S5. Comparison of GSEA output: cell motility geneset (GO:0048870) expression levels in tumors derived from KR158 cells cultured with serum versus GL261 cells. Table S6. Comparison of GSEA output: cell motility geneset (GO:0048870) expression levels in tumors derived from KR158 cells cultured with serum versus CT-2A cells. Table S7. Comparison of GSEA output: ECM receptor interaction geneset (KEGG mmu04512) expression levels in tumors derived from KR158 cells cultured with serum versus GL261 cells. Table S8. Comparison of GSEA output: ECM receptor interaction geneset (KEGG mmu04512) expression levels in tumors derived from KR158 cells cultured with serum versus CT-2A cells. Table S9. Comparison of GSEA output: SCC-related geneset [19,20] expression levels in tumors derived from KR158 cells cultured with serum versus GL261 cells. Table S10. Comparison of GSEA output: SCC-related geneset [19,20] expression levels in tumors derived from KR158 cells cultured with serum versus CT-2A cells. Table S11. Comparison of GSEA output: stemness geneset [38] expression levels in tumors derived from KR158 cells cultured in the gliomasphere assay versus KR158 cells expanded as monolayer in serum-containing conditions. Table S12. Comparison of GSEA output: stemness geneset [39] expression levels in tumors derived from KR158 cells cultured in the gliomasphere assay versus KR158 cells expanded as monolayer in serum-containing conditions. Table S13. List of pathways upregulated from the stemness gene network identified in KR158 cells cultured in the gliomasphere assay compared to adherent serum-containing conditions. Table S14. Comparison of GSEA output (gliomasphere assay): stemness geneset [38] expression levels in SCCs versus FCCs. Table S15. Comparison of GSEA output (gliomasphere assay): stemness geneset [39] expression levels in SCCs versus FCCs. Table S16. Comparison of GSEA output (gliomasphere assay): SCC-related geneset [19,20] expression levels in SCCs versus FCCs. Table S17. Comparison of GSEA output (gliomasphere assay): migration geneset (GO:0016477) expression levels in SCCs versus FCCs. Table S18. Comparison of GSEA output (gliomasphere assay): cell motility geneset (GO:0048870) expression levels in SCCs versus FCCs. Table S19. Comparison of GSEA output (gliomasphere assay): ECM receptor interaction geneset (KEGG mmu04512) expression levels in SCCs versus FCCs. Table S20. Comparison of GSEA output (gliomasphere assay): lipid catabolic process geneset (GO:0016042) expression levels in SCCs versus FCCs. Table S21. Comparison of GSEA output (gliomasphere assay): cellular response to lipid geneset (GO:0071396) expression levels in SCCs versus FCCs. Table S22. Comparison of GSEA output: stemness geneset [38] expression levels in tumors derived from SCCs versus tumors formed by FCCs, with cells expanded in the gliomasphere assay. Table S23. Comparison of GSEA output: migration geneset (GO:0016477) expression levels in tumors derived from SCCs versus tumors formed by FCCs, with cells expanded in the gliomasphere assay. Table S24. Comparison of GSEA output: cell motility geneset (GO:0048870) expression levels in tumors derived from SCCs versus tumors formed by FCCs, with cells expanded in the gliomasphere assay. Table S25. Comparison of GSEA output: ECM receptor interaction geneset (KEGG mmu04512) expression levels in tumors derived from SCCs versus tumors formed by FCCs, with cells expanded in the gliomasphere assay. Table S26. Comparison of GSEA output: cellular response to lipid geneset (GO:0071396) expression levels in tumors derived from SCCs versus tumors formed by FCCs, with cells expanded in the gliomasphere assay. Supplemental Figure S1. H&E labeling showing necrotic foci (*) in tumors derived from cells expanded in the gliomapshere assay (A) or adherent in presence of serum (B). Supplemental Figure S2. Electropherograms of the 19 STR markers and table indicating 100% match between KR158 cells cultured in both assays. Supplemental Figure S3. H&E labeling showing necrotic areas (*) in tumors generated by SCCs. Supplemental Figure S4. H&E stain displaying necrosis (*) in FCC tumors. Supplemental Figure S5. Presence of SCCs in gliomasphere cultures of CT-2A. (A) Brightfield image of spheres formed by CT-2A cells expanded in serum-free media supplemented with EGF and FGF2. (B) Flow cytometry gating strategy to identify and isolate SCCs vs. FCCs.
