# Supplementary figures and images for: KR158 Spheres Harboring Slow-Cycling Cells Recapitulate High-Grade Glioma Features in an Immunocompetent System
Source: Cells. 2024 May 29;13(11):938. doi: 10.3390/cells13110938 (PMC11171638; doi:10.3390/cells13110938)

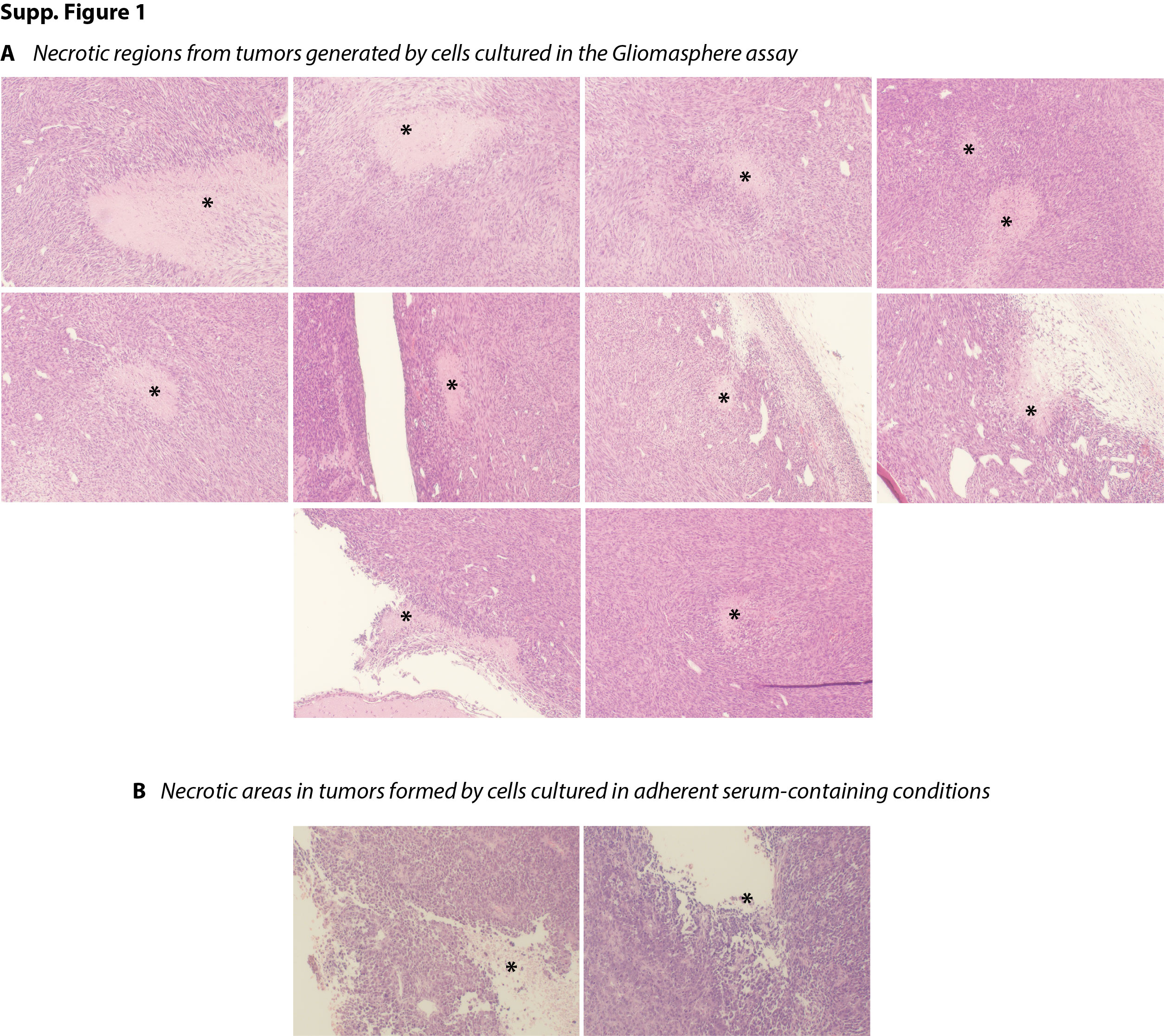

Supplement: Supplementary file 1 [file cells-13-00938-s001.zip › Supp. Fig.S1.jpg]

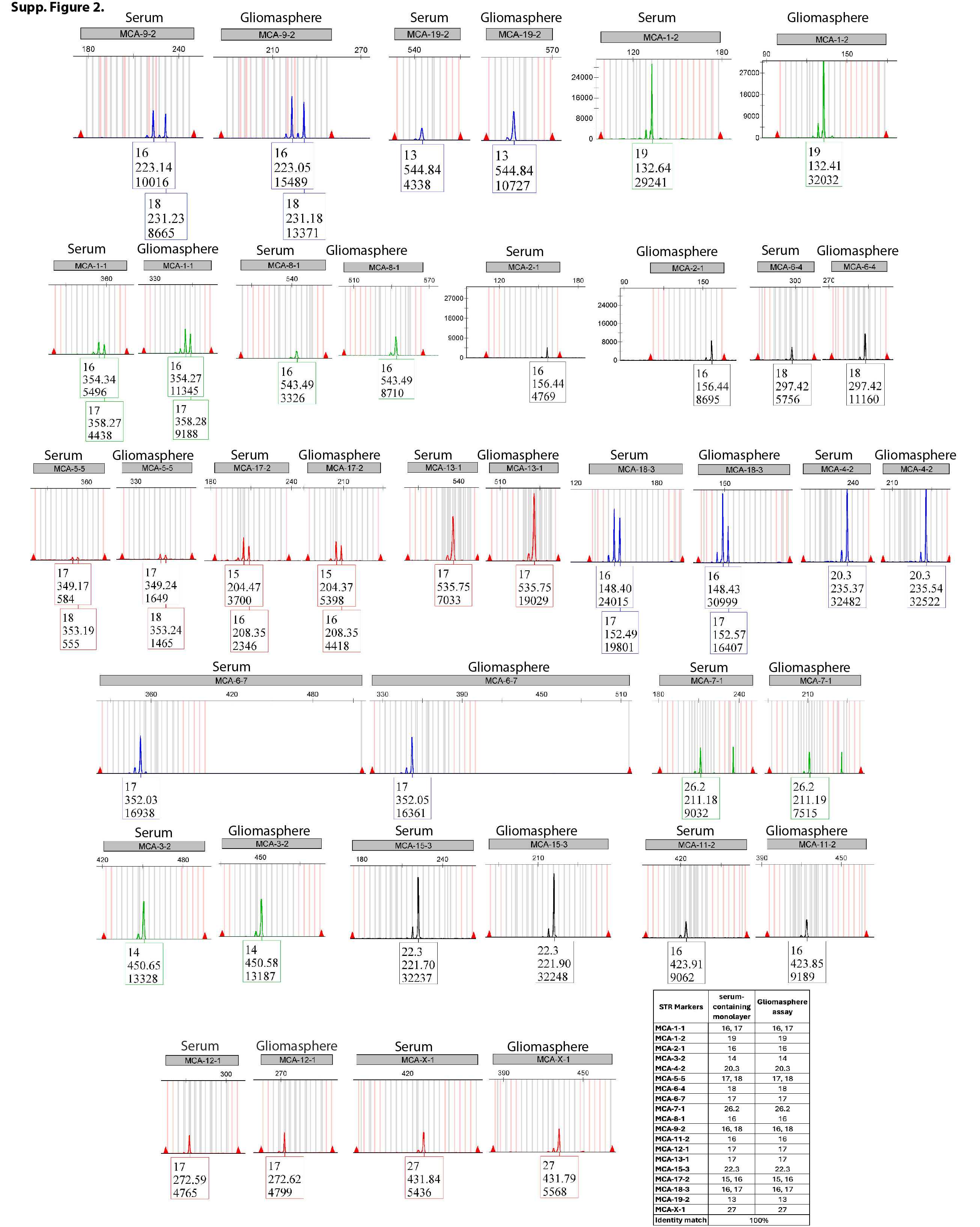

Supplement: Supplementary file 1 [file cells-13-00938-s001.zip › Supp. Fig.S2.jpg]

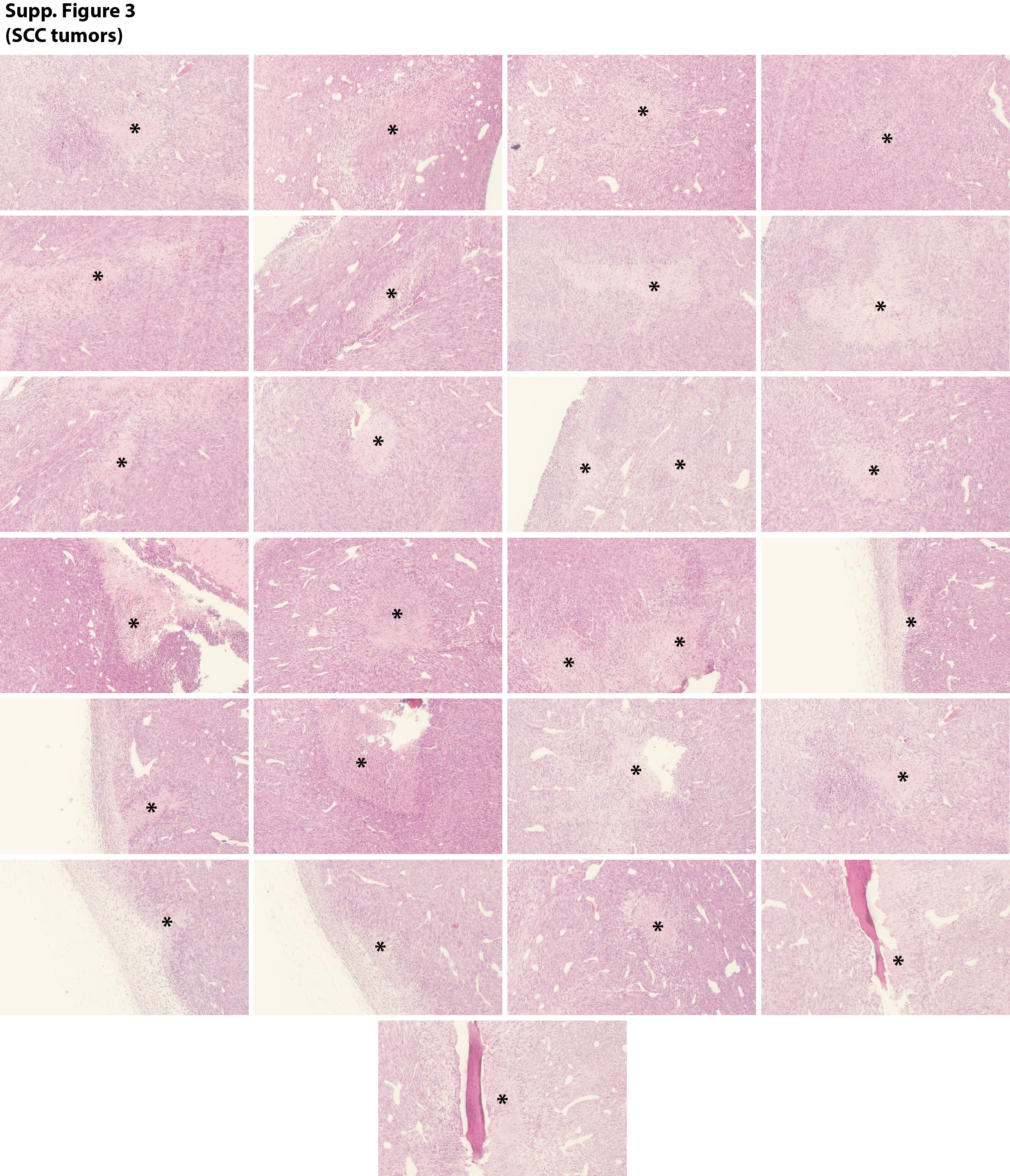

Supplement: Supplementary file 1 [file cells-13-00938-s001.zip › Supp. Fig.S3.jpg]

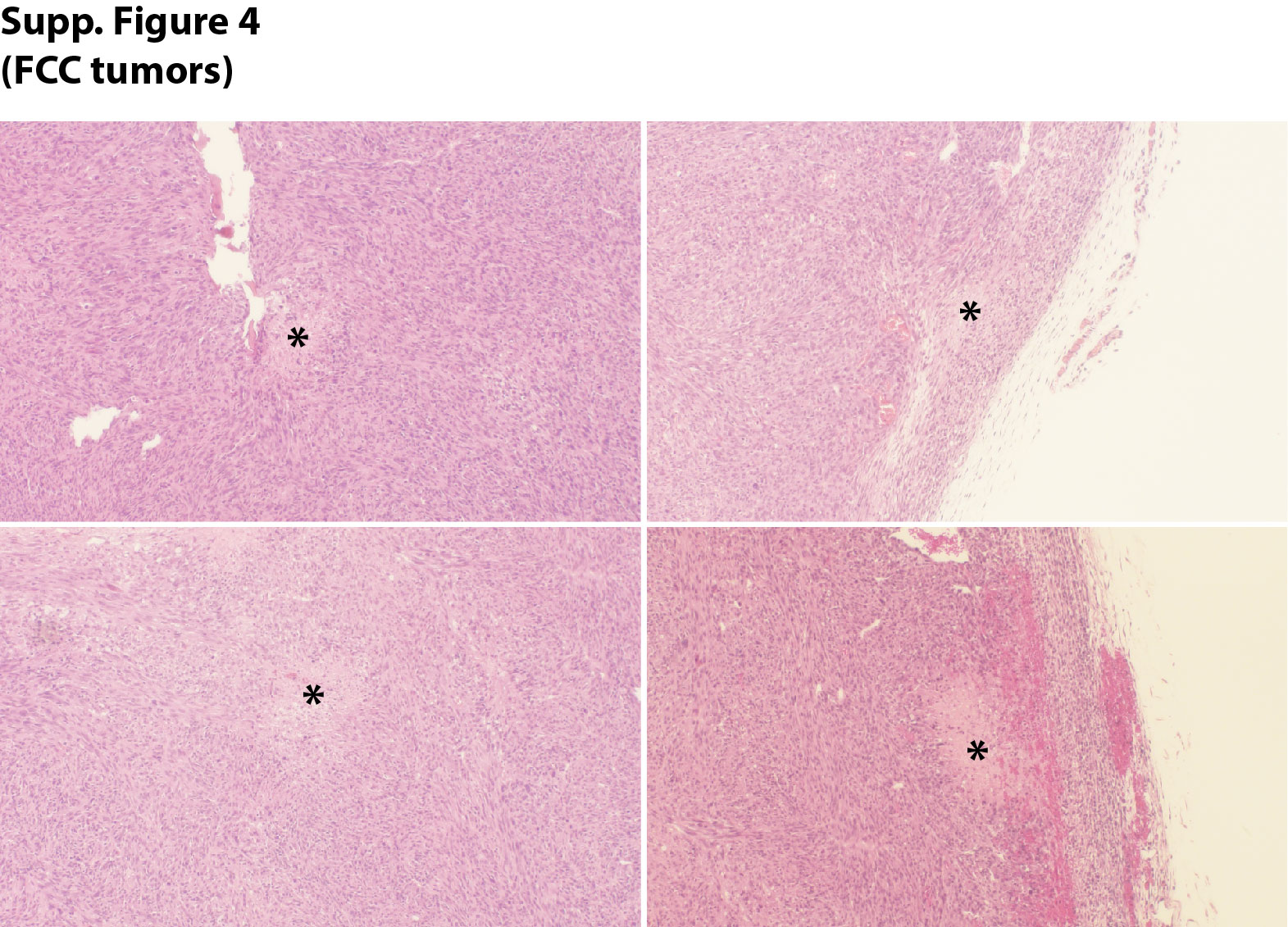

Supplement: Supplementary file 1 [file cells-13-00938-s001.zip › Supp. Fig.S4.jpg]

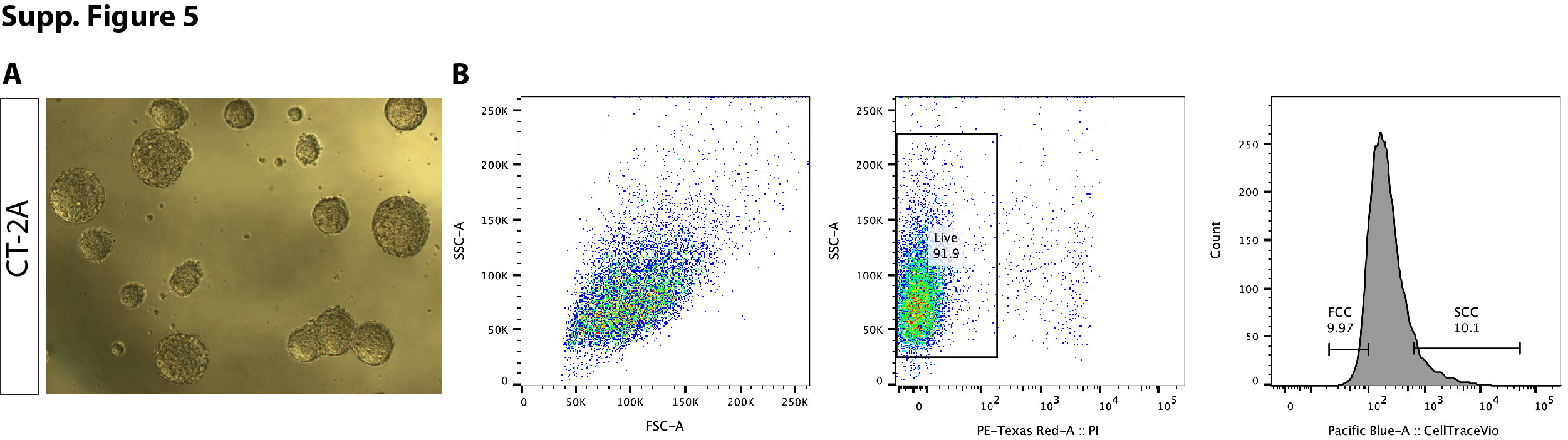

Supplement: Supplementary file 1 [file cells-13-00938-s001.zip › Supp. Fig.S5.jpg]
